# Intralesional Alteplase as an Adjunct to Percutaneous Drainage for a Large Iliopsoas Muscle Hematoma in a Patient With Von Willebrand Disease: A Case Report

**DOI:** 10.7759/cureus.95920

**Published:** 2025-11-01

**Authors:** Estefania Gomez-Charnichart, Rodrigo Bravo-Garcia, Luis Adrián Álvarez-Lozada, Gabriel Maldonado-Rangel, Miguel Alejandro Campos-Bocardo, Adrian Argüello-Piña, Benjamin Moya-Leal, Eduardo Flores-Villalba

**Affiliations:** 1 Department of Surgery, Institute for Social Security and Services for State Workers (ISSSTE) Hospital Regional de Monterrey, Monterrey, MEX; 2 Department of Human Anatomy, Universidad Autónoma de Nuevo León, School of Medicine, Monterrey, MEX; 3 Department of Interventional Radiology, Institute for Social Security and Services for State Workers (ISSSTE) Hospital Regional de Monterrey, Monterrey, MEX; 4 Department of Hematology, Institute for Social Security and Services for State Workers (ISSSTE) Hospital Regional de Monterrey, Monterrey, MEX

**Keywords:** alteplase, femoral neuropathy, iliopsoas muscle hematoma, percutaneous drainage, von willebrand disease

## Abstract

We present the case of a 32-year-old woman with type III von Willebrand disease who, after a chiropractic manipulation, developed a large iliopsoas muscle hematoma with femoral neuropathy. Given the lack of recombinant factor VIII at our institution, we opted for a minimally invasive, interdisciplinary approach. The patient was clinically stable and fully adherent to the proposed interdisciplinary management plan. A percutaneous drainage was placed and supplemented with intralesional alteplase irrigations, which allowed for a progressive reduction of the hematoma, resolution of the neuropathy, and the patient's complete recovery. The patient was discharged with minimal residual weakness, and at six weeks, imaging showed near-complete resolution of the collection and full return to daily activities. This case demonstrates the success of combining percutaneous drainage with fibrinolytic agents as an effective and safe alternative to conventional surgical management in patients with severe coagulopathies.

## Introduction

Von Willebrand disease (vWD) is the most common inherited bleeding disorder and results from quantitative or qualitative defects of von Willebrand factor (vWF), a protein secreted by the vascular endothelium that mediates platelet adhesion and stabilizes factor VIII in the coagulation cascade. The hereditary form may present with easy bruising, mucosal bleeding (such as epistaxis or gingival bleeding), prolonged hemorrhage after minor trauma or dental procedures, and, in women, menorrhagia. Hematuria and hematochezia have also been reported [1].

Retroperitoneal hematomas are uncommon but potentially life-threatening, defined as bleeding into the retroperitoneal cavity. This space is divided into three anatomical zones: central-medial (zone 1), perirenal (zone 2), and pelvic (zone 3) [2]. They may occur spontaneously or after trauma, with blunt injuries being more frequent. Clinical manifestations depend on blood loss and may progress to hemorrhagic shock. Computed tomography (CT) is the gold standard for diagnosis [3].

Management is challenging due to the deep location and the risk of uncontrollable bleeding, especially in patients with congenital coagulopathies. While surgical exploration is usually reserved for expanding or pulsatile hematomas and those involving zones 1 or 3, supportive therapy and factor replacement remain critical [3]. However, specific treatments such as recombinant factor VIIa or vWF concentrates are costly and not always available, which complicates timely intervention and increases morbidity and mortality [2]. This case underscores the importance of a multidisciplinary approach and a unique alternative to treatment with percutaneous drainage and alteplase irrigation when access to recombinant factors is limited.

## Case presentation

We present the case of a 32-year-old woman who was treated in June 2025 at the Institute for Social Security and Services for State Workers (ISSSTE) Regional Hospital in Monterrey, Nuevo León, Mexico, for a history of vWD. She had a history of hypermenorrhea and irregular menstrual cycles, which frequently required blood transfusions.

**Figure 1:**
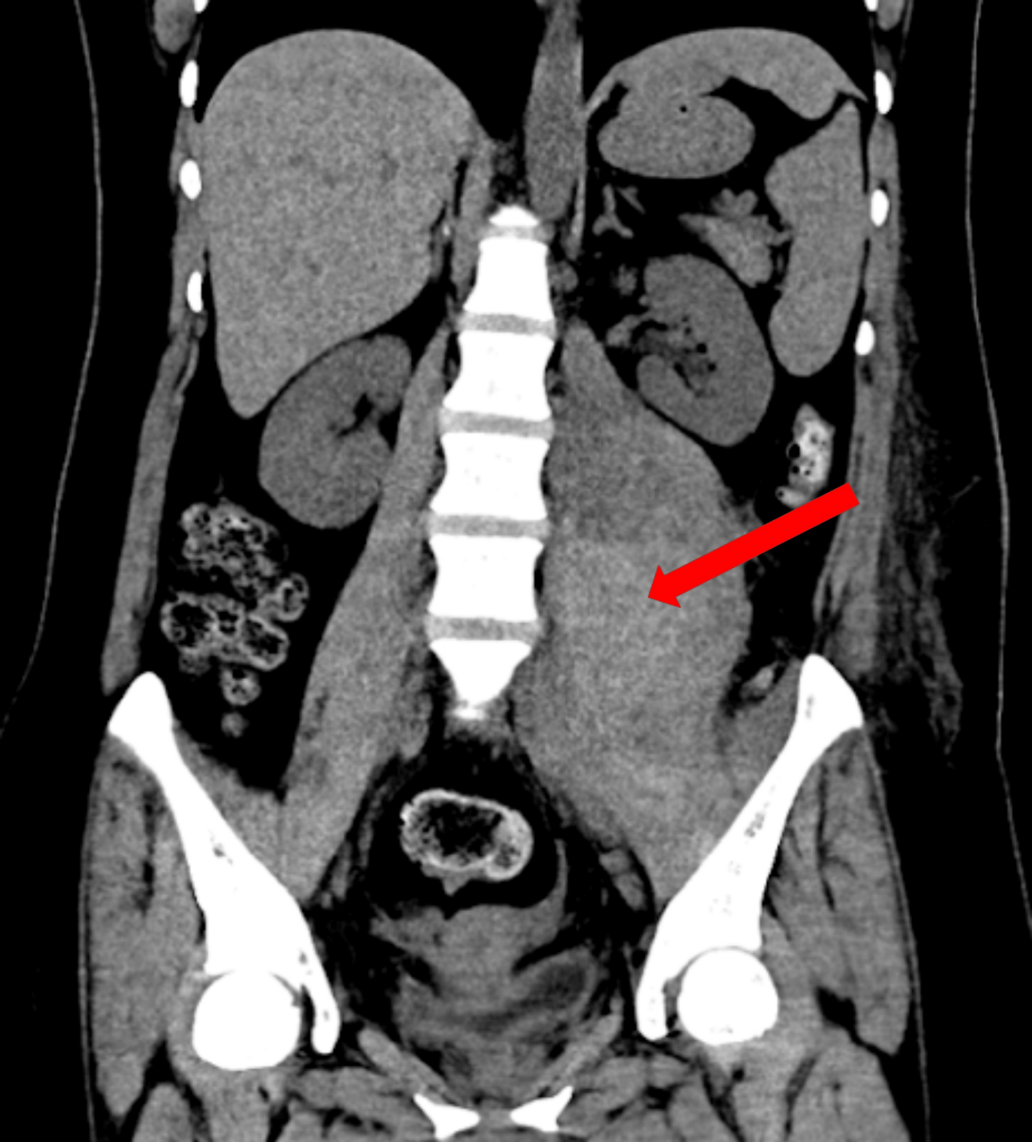
The first CT coronal reconstruction shows a heterogeneous image in the left iliopsoas muscle, consistent with a hematoma, with an estimated volume of 700 cc.

Following a chiropractic consultation, the patient presented with a large left inguinal hematoma, femoral neuropathy, and pain 10/10 on the Numeric Pain Rating Scale (NPRS). A CT scan revealed a 689 cc (Figure 1) hematoma in the left iliopsoas muscle. She was admitted, and an interdisciplinary consultation with hematology confirmed a diagnosis of type III vWD. Given the lack of recombinant factor VIII at our hospital, initial treatment included transnasal desmopressin, tranexamic acid, cryoprecipitates, and fresh-frozen plasma (FFP) to stabilize her hemoglobin levels.

After stabilization, the general surgery, hematology, and interventional radiology (IR) departments made an interdisciplinary decision to perform a minimally invasive approach due to the high risk of intraoperative hemorrhage and the patient's preference.

In the first week, a 12 Fr pigtail percutaneous drainage catheter (PDC) was placed by the IR service. The following week, due to persistent pain and no improvement in femoral neuropathy, a collective decision was made to perform an intralesional irrigation with alteplase through the PDC. This involved irrigating with a prepared solution containing 3 mg of alteplase from a 50 mg/50 mL manufacturer dilution.

**Figure 2:**
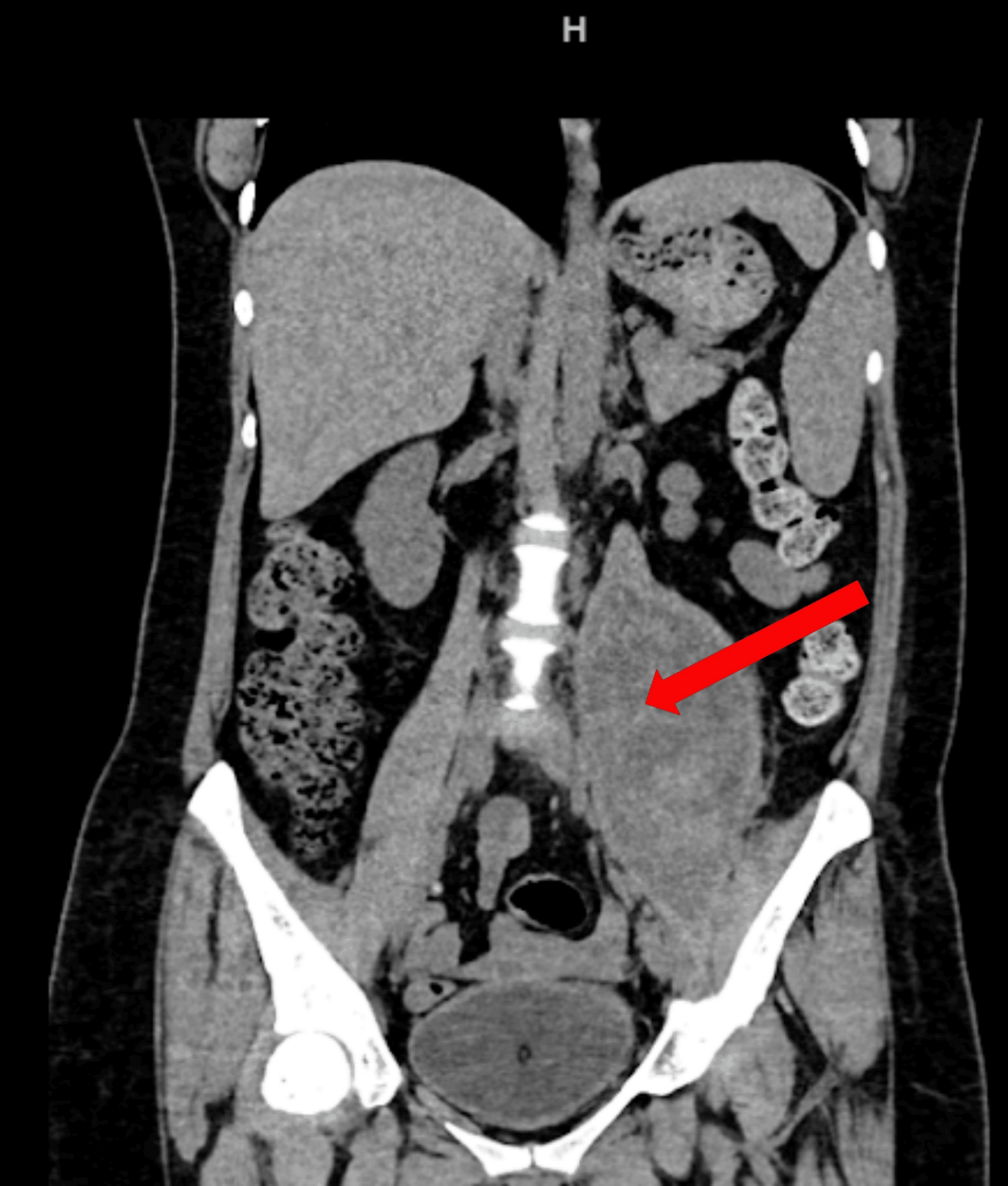
The second CT coronal reconstruction shows a decrease in the hematoma within the left iliopsoas muscle, with an estimated volume of 350 cc.

By the third week, a control CT showed the hematoma volume decreased to 352 cc (Figure 2), leading to a notable clinical improvement. Cryoprecipitates and FFP were discontinued. However, the patient developed macroscopic hematuria due to a urinary tract infection (UTI) during the third week, requiring a transfusion and a restart of cryoprecipitates and FFP. After resolution of the infection and a blood transfusion, her hemoglobin levels returned to normal, confirming the suspected cause of the decrease in hemoglobin levels. Also in this week, a second alteplase irrigation was performed using a lower concentration. For this second irrigation, a 2 mg solution was prepared.

**Figure 3:**
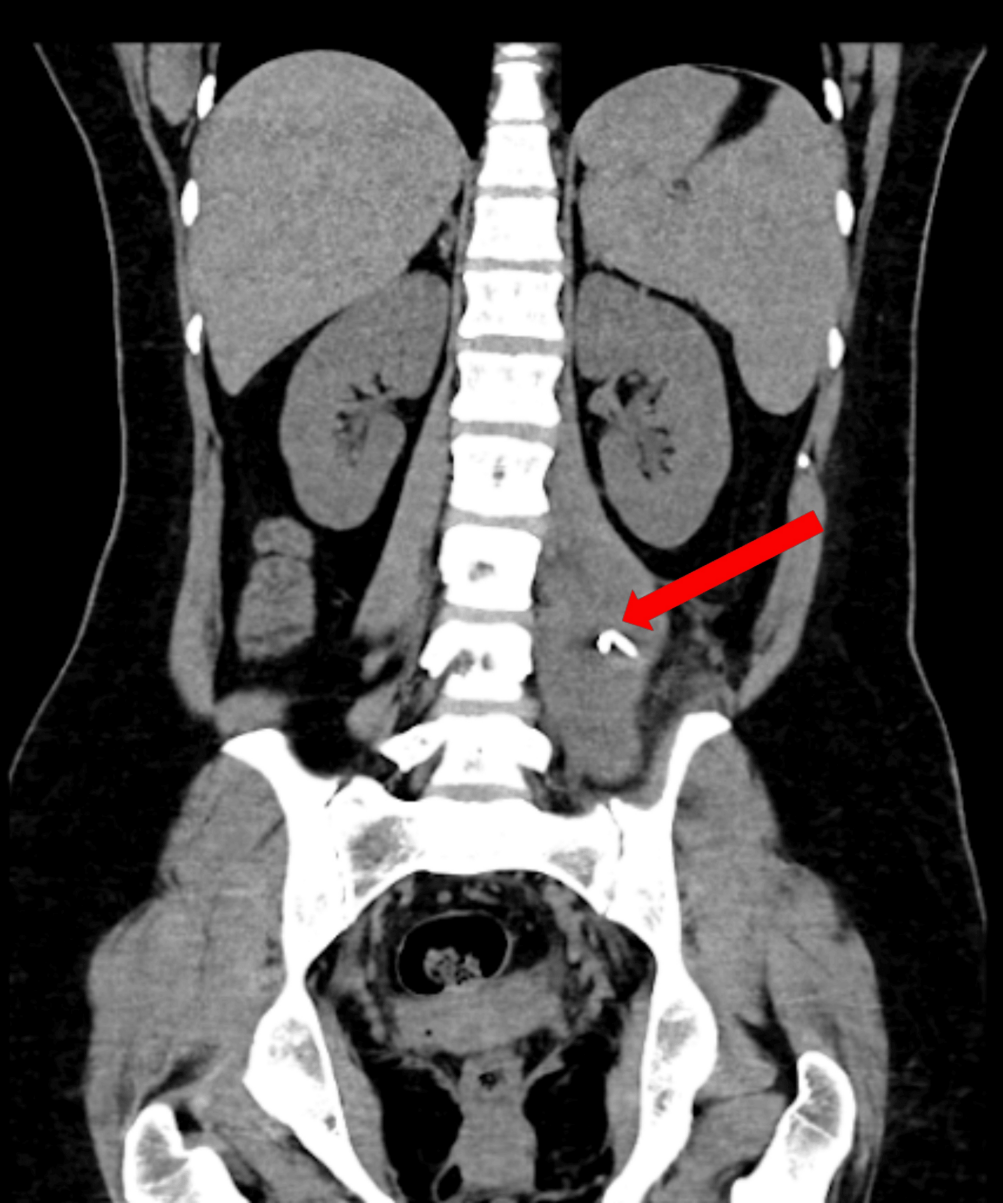
The third CT coronal reconstruction shows a marked reduction in the size of the hematoma, with an estimated volume of 200 cc, and demonstrates the presence of a 12 Fr "pigtail" catheter within it.

In the fourth week, a CT scan showed a further decrease to 260 cc (Figure 3). The patient reported resolution of pain, improved leg strength (4/5), and limited paresthesias, and she was able to ambulate. Her hemoglobin level remained stable, allowing the PDC to be removed and her discharge despite the residual hematoma. Outpatient follow-up was scheduled.

Two weeks after discharge, the patient was in good condition and ambulating with only slight weakness. An MRI conducted at six weeks revealed a residual collection of 43 cc. Her blood work showed stable hemoglobin, a vWF of <10%, and a factor VIII of 70%. She had returned to daily activities and was referred to rehabilitation.

## Discussion

Iliopsoas hematomas in patients with vWD are exceptionally rare. Standard management usually involves the administration of recombinant factor VIII or vWF concentrates. While iliopsoas hematomas are more frequently described in patients with hemophilia, particularly in severe forms, their occurrence in vWD, especially type III, is extremely uncommon.

A comparable case reported in the literature described a young male with vWD type III who developed a spontaneous iliopsoas hematoma and responded favorably to standard factor replacement therapy [4]. Another report documented management with vWF replacement; however, percutaneous drainage was required because the hematoma did not resolve conservatively [5]. These cases highlight the critical role of factor replacement in successful management. In contrast, our case is distinctive because factor VIII/vWF concentrates were unavailable, necessitating an unconventional yet effective strategy consisting of percutaneous drainage combined with intralesional alteplase, a therapeutic approach not previously described in this patient population.

Historical management of iliopsoas hematomas in bleeding disorders generally centers on factor replacement with or without surgical decompression in cases with neurological compromise. Hematomas more commonly occur in hemophilia or in anticoagulated patients, with management strategies ranging from conservative treatment (rest and replacement therapy) to open surgical evacuation in more severe or neurologically impaired cases [6]. There is, however, no precedent in the literature for intralesional fibrinolytic therapy in this patient population.

In our patient, the clinical course was favorable, with hematoma dimensions decreasing from 22.3 × 8.7 × 6.8 cm (volume 689 cc) to 13.2 × 6.5 × 3.6 cm (volume 260 cc) within four weeks, while hemoglobin levels remained stable, and partial recovery of strength and mobility in the left thigh was achieved. Percutaneous drainage is a well-established therapeutic option; however, in the absence of supplementation with the deficient factor, its recurrence rate has been reported to be approximately 13% [7]. Remarkably, in this case, further reduction of the hematoma to 7.8 × 4.0 × 2.7 cm (approximate volume 43 cc) was achieved six weeks after discharge. This suggests that the addition of intralesional alteplase to percutaneous drainage, even in the absence of factor VIII, may reduce complications and the recurrence of iliopsoas hematomas.

Rehabilitation remains an essential component of care in these patients, particularly when compressive femoral neuropathy is present, since weakness and/or paresthesias may persist even after hematoma resolution. Early involvement of rehabilitation services is crucial for restoring mobility and strength, as indicated in this case [6].

Nevertheless, caution is warranted. The use of alteplase in patients with inherited bleeding disorders theoretically carries a significant risk of hemorrhagic complications, and the safety of this approach cannot be generalized based on a single case. Larger case series or prospective studies would be necessary to validate the efficacy and safety of intralesional fibrinolysis in this context.

This case underscores the importance of a multidisciplinary approach and illustrates that, in resource-limited settings where specific factor concentrates are unavailable, innovative strategies such as percutaneous drainage combined with intralesional alteplase may provide a viable therapeutic alternative.

## Conclusions

Iliopsoas hematomas in patients with vWD type III are extremely rare and typically managed with factor replacement therapy, sometimes combined with surgical or percutaneous drainage. Our case is unique in demonstrating that, in the absence of factor VIII/vWF concentrates, the combination of percutaneous drainage with intralesional alteplase resulted in significant hematoma resolution, clinical improvement, and functional recovery. While this approach may represent a promising therapeutic alternative in resource-limited settings, its use should be undertaken with caution, given the potential risk of bleeding. Further reports and larger studies are needed to establish the safety, efficacy, and long-term outcomes of intralesional fibrinolytic therapy in this patient population.

## References

[1] Nichols WL, Hultin MB, James AH (2008). von Willebrand disease (VWD): evidence-based diagnosis and management guidelines, the National Heart, Lung, and Blood Institute (NHLBI) Expert Panel report (USA). Haemophilia.

[2] Mondie C, Maguire NJ, Rentea RM (2024). Retroperitoneal hematoma. StatPearls [Internet].

[3] Sabih A, Babiker HM (2023). Von Willebrand disease. StatPearls [Internet].

[4] Keikhaei B, Soltani Shirazi A (2011). Spontaneous iliopsoas muscle hematoma in a patient with von Willebrand disease: a case report. J Med Case Rep.

[5] Eby CS, Caracioni AA, Badar S, Joist JH (2002). Massive retroperitoneal pseudotumour in a patient with type 3 von Willebrand disease. Haemophilia.

[6] Basheer A, Jain R, Anton T, Rock J (2013). Bilateral iliopsoas hematoma: case report and literature review. Surg Neurol Int.

[7] Rodriguez-Merchan EC (2020). Hemophilic pseudotumors: diagnosis and management. Arch Bone Jt Surg.

